# Teleneurosurgery: Outcome of Mild Head Injury Patients Managed in Non-Neurosurgical Centre in the State of Johor

**DOI:** 10.21315/mjms2018.25.2.10

**Published:** 2018-04-27

**Authors:** Mohd Syahiran Mohd Sidek, Johari Adnan Siregar, Abdul Rahman Izani Ghani, Zamzuri Idris

**Affiliations:** 1 Department of Neurosciences, School of Medical Sciences, Universiti Sains Malaysia, 16150 Kubang Kerian, Kelantan, Malaysia; 2 Center for Neuroscience Services and Research, Universiti Sains Malaysia, 16150 Kubang Kerian, Kelantan, Malaysia; 3 Department of Neurosurgery, Hospital Sultanah Aminah Johor Bahru, 80100 JohorBharu, Malaysia; 4 Department of Neurosurgery, Hospital Kuala Lumpur, 50586, Wilayah Persekutuan Kuala Lumpur, Malaysia

**Keywords:** neurosurgical unit, general surgical unit, delay transfer, mild head injury

## Abstract

**Background:**

With teleneurosurgery, more patients with head injury are managed in the primary hospital under the care of general surgical unit. Growing concerns regarding the safety and outcome of these patients are valid and need to be addressed.

**Method:**

This study is to evaluate the outcome of patients with mild head injury which were managed in non-neurosurgical centres with the help of teleneurosurgery. The study recruits samples from five primary hospitals utilising teleneurosurgery for neurosurgical consultations in managing mild head injury cases in Johor state. Two main outcomes were noted; favourable and unfavourable, with a follow up review of the Glasgow Outcome Scale (GOS) at 3 and 6 months.

**Results:**

Total of 359 samples were recruited with a total of 11 (3.06%) patients have an unfavourable. no significant difference in GOS at 3 and 6 months for patient in the unfavourable group (*P* = 0.368).

**Conclusion:**

In this study we have found no significant factors affecting the outcome of mild head injury patients managed in non-neurosurgical centres in Johor state using the help of teleneurosurgery.

## Introduction

Neurosurgical services are not widely available in all hospitals in Malaysia. As to overcome this shortage, neurosurgical services are provided at centrally located hospitals. Patients with neurosurgical related problems which present themselves to centres without neurosurgical services, need to be referred via telephone conversation without images or video conferencing technique (1). The emergence of telemedicine services in recent years has further improvised the neurosurgical services.

Since 2006, telemedicine in neurosurgery or teleneurosurgery has been widely used for transmission of clinical data and images throughout Malaysia (2). Many patient suffers from traumatic head injury were being evaluated in hospitals with no neurosurgical services and are successfully managed in the primary hospital with the help of teleneurosurgery. In a recent local study by Hassan et al. (2), noted that 37% of transfer was avoided and patients were best kept in their primary hospitals by utilising teleneurosurgery. The swift advances in information technology exchange have also reshaped the way we practice medicine. With the help of teleneurosurgery, there is global scale communication and transmission of data which precludes unnecessary patients transfer (2, 5, 6), thus further reduces the costs for patients and medical providers.

Glasgow Coma Scale (GCS) has become the gold standard tool for assessment of patient with traumatic head injury since its landmark paper publication by Teasdale and Jennett more than 40 years ago (5, 7). With computerised tomography (CT) scans widely available nowadays, a combination of both GCS and CT scan findings should be taken into consideration in stratifying the severity of head injury and the treatment strategy (8). Traumatic head injury could be divided into three categories: i) mild head injury [GCS: 13–15], ii) moderate head injury [GCS: 9–12], and iii) severe head injury [GCS: 3–8] (9–11). The practice of managing head injury patient harbouring a non-surgical lesion in the primary hospital under the care of general surgical unit (GSU) is considered as safe, economical and acceptable (5). The population of interest in this study were patients who suffer from mild head injury, which were kept in the primary hospital with remote consultation using the help of teleneurosurgery.

As more patients are kept in the primary hospitals, without immediate neurosurgical services, there are growing concerns regarding the safety and outcome of these patients which are co-managed remotely with the centralised neurosurgical team via teleneurosurgery. This paper is to evaluate the outcome of patients with mild head injury who was managed in a non-neurosurgical centre with the help of teleneurosurgery.

## Methodology

The study was a cross-sectional observational study which was conducted in the period of 16 months from the month of June 2015 through September 2016. The sample size of this study was calculated based on the previous study by Klein et al. (13). Based on the study quoting a 97% of good outcome (13) of mild head injury patient, with an expected 2% difference in proportion of the good outcome from the reference, the sample size calculated was 280 samples.

A total of five peripheral hospitals without neurosurgical services in the state of Johor were enrolled in this study. The five hospitals includes: i) Hospital Sultan Ismail (HSI), ii) Hospital Muar, iii) Hospital Segamat, iv) Hospital Kluang, and v) Hospital Batu Pahat. Head injury cases seen in these peripheral hospitals were referred to a central neurosurgical unit (NSU) for the state of Johor, which is located in Hospital Sultanah Aminah Johor Bahru (HSAJB). Subjects were identified using the database from the official Neurosurgery HSAJB department e-mail and teleconsultation. Both departmental e-mail and teleconsultation were considered as teleneurosurgery.

Patients with suspected blunt traumatic brain injury that meets the criteria based on Canadian CT Head Rule (11, 14) as portrayed in Table 1 had an unenhanced computerised tomography (CT) of the head on presentation. The patients were attended by the general surgeon in the primary hospital. The decision for an immediate transfer of the patients to the NSU or to be managed in the primary hospital under the care of GSU was made after a phone consultation between both parties. Clinical data and CT images were conveyed via teleneurosurgery. High risk patients were transferred to HSAJB under the care of NSU. Low risk patients who were not transferred, were admitted to the GSU in the primary hospital and neurological evaluation was performed by the surgical staff. A repeat CT scan was performed for patients with positive CT findings within 24 h to 48 h after admission or earlier in case of neurological deterioration. In case of neurological deterioration or worsening bleed, the patients were transferred to the NSU in HSAJB for further intervention or observation. Low risk traumatic brain injuries were defined as no intracranial bleed (ICB), solitary brain contusion < 1 cm in diameter, minimal subdural hematoma < 0.5 cm in maximal width, small subarachnoid hemorrhage (SAH) and no signs of mass effect (13). The decision for placement of patients either to be kept in the primary hospital under GSU or to be transferred to NSU was based on the criteria mentioned above.

Inclusion criteria for this study were: i) mild head injury with the Glasgow coma scale (GCS) ≥ 13 (9–11, 13) ii) clear history of trauma, iii) age ≥ 18 years old, iv) the first referral must be within 24 h of initial trauma, v) patients were decided by the neurosurgical team to be managed in the primary hospital under the care of general surgical unit (GSU). Exclusion criteria includes: i) incomplete referral; either clinical data or images, including poor image quality (2), ii) referral made after 24 h of the initial trauma, iii) GCS ≤ 12, iv) other mode of referrals such as via multimedia messaging services (MMS) (2), v) age ≤ 17 years old, vi) polytraumatised patients.

The primary end point of this study is to determine the outcome of the patient managed in GSU at the time of discharge. A favourable outcome was defined; by discharge from the primary hospital with the similar or a better GCS score from the initial presentation. An unfavourable outcome was considered if there was a need for a delayed transfer to NSU, discharge from the primary hospital with a lower GCS score comparatively to the initial presentation or death. The outcome of these patients were collected by the primary investigator via phone call to the peripheral hospital. Patients which were discharge either from GSU or NSU after a delayed transfer were seen in the neurosurgical clinic in 3 and 6 months’ time. The condition of the patient during the follow up at 3 and 6 months’ time (15) were recorded and the Glasgow Outcome Scale (GOS) (10, 16) was determined by the attending medical officer. The attending medical officers were trained beforehand, in answering the GOS questionnaire in order to reduce observers’ bias. Secondary endpoint of the study was to determine and compare the GOS at 3 and 6 months in the favourable and unfavourable group.

Age, sex, ethnicity, radiological diagnosis, GCS on admission and upon discharge, duration of stay and types of referral were collected to describe the variability of the study population. Statistical analysis was performed using Statistical Package for Social Sciences (SPSS) version 22 software. Demographics were expressed in table form using mean and standard deviation (SD) for numerical variables and numbers and percentages for categorical variables. McNemar test was used in order to determine the difference in the GOS of both favourable and unfavourable group at 3 and 6 months. Outcome predictors were analysed using univariate logistic regression analysis as to give the crude and odd ratio. Statistical significance was considered when *P* was ≤ 0.05.

Study proposal was sent for approval from Malaysian Medical Research and Ethics Committee (MREC). A letter of approval of the study is shown as attached [NMRR ID: NMRR-15-1895-25648].

## Results

Between the period of June 1, 2015 and September 30, 2016, 395 patients were referred to NSU HSAJB via teleneurosurgery with the GCS of ≥ 13. A total number of 359 (*n*) patients were enrolled in this study and 36 patients were excluded as it does not satisfy the inclusion and exclusion criteria. Five patients were excluded as having polytrauma, 20 patients were excluded as they were late referral; > 24 h after initial trauma, and the rest were because of incomplete referral with poor image quality.

Table 2 summarises the demographics of the population studied. Out of 359 (*n*) patients, 277 (77.2%) were male and 82 (22.8%) were female. The population ranges from 18 to 88 years old with the mean (SD) of 45.39 (20.23). Malay comprises the majority of patients, with a total of 217 (60.45%) followed by Chinese, 91 (25.35%). The mode of referral for teleneurosurgery was almost equal between official departmental e-mail, 180(50.14%) and teleconference, 179 (49.84%). Hospital Muar leads the number of cases referred using teleneurosurgery, 105 (29.25%), followed by Hospital Batu Pahat, 98 (27.3%), Hospital Sultan Ismail, 83 (23.12%), Hospital Segamat, 38 (10.58) and Hospital Kluang, 35 (9.75%). Mean (SD) GCS on referral was 14.52 (0.72), with 234 (65.18%) of the population have a full GCS upon referral. Mean (SD) GCS upon discharge was 14.89 (0.78) with one reported death in this series. Radiologically, 115 (32.0%) of the population have sustained subdural hemorrhage (SDH), followed by no evidence of intracranial bleed (ICB) 96 (26.7%); hemorrhagic contusion 63 (17.5%); subarachnoid hemorrhage (SAH) 43 (12.0%), and extradural hemorrhage (EDH) 42 (11.7%). The mean (SD) duration of stay was 2.16 (1.19) days.

Ten (2.79%) patients had a delay transfer to NSU HSAJB for further management. Out of the 10 delay transfer, 6 (60%) needed surgical intervention and 4 (40%) did not deemed any. The cases needing surgical intervention includes; two cases of EDH, one case of SDH, one contusion case, and two newly developed EDH case on repeat CT brain with the initial CT shows SAH and another showing no ICB, respectively. One death was recorded from the SDH case needing surgical intervention. Except for the two newly found EDH on repeat CT scan, all the operated cases were brought over to NSU HSAJB with the expansion of the initial ICB. The other four delay transfer cases also shows expansion of the initial ICB, otherwise was managed conservatively as having good GCS upon review.

Out of the study population (*n* = 359), 348 (96.94%) patients have favourable outcome with 11 (3.06%) have unfavourable outcome. Ten out of the 11 unfavourable outcome was due to the need of a delay transfer, one of the patient was in the unfavourable group due to a lower documented GCS: 13 upon discharge comparatively to the admission GCS: 14. A total number of 319 (88.86%) and 311 (86.63%) of the population have a GOS of 5: good recovery, at 3 and 6 months, respectively.

There were a total of two recorded deaths in the period of the study. One death was after transfer to NSU for surgical intervention of an acute SDH expansion. This was a case of a 34 years old man who was involved in a motor vehicle accident. He sustained mild head injury with the initial GCS on presentation of 14. Initial CT brain reveals a thin acute subdural component over the right convexity with no mass effect. He was managed in the GSU according to the head injury protocol with the help of teleneurosurgery. After 24 h of presentation, his GCS had dropped to 8 and repeated CT brain reveals expansion of the SDH. He was transferred to NSU HSAJB and underwent decompressive craniectomy and clot evacuation. His condition remains poor post-surgery and eventually succumbs on day 5 post-surgery. The other death was recorded at home, 4 months after the initial trauma, and was attributed due to old age. This particular case was excluded from the study.

On outcome crosstabulation, as depicted in Table 3; 9 (3.25%) of the male population have an unfavourable outcome while only 2 (2.24%) have an unfavourable outcome in the female population. Three (1.38%) of the Malay and 4 (4.40%) of the Chinese population have an unfavourable outcome, respectively. Almost a similar percentage was noted in the unfavourable outcome group for those presented with a GCS of 13, 6.12% (3) and GCS 14, 6.58% (5). Radiologically, 2 (4.8%) out of 40 (95.2%) of the EDH patients have an unfavourable outcome, while in the SDH, 3 (2.6%) out of 112 (97.4%) have an unfavourable outcome. For patients with contusional bleed, 4 (6.3%) patients have an unfavourable outcome whilst 1 (2.3%) patient with SAH also has an unfavourable outcome.

McNemar test was used in order to determine whether there is any significant difference in the GOS at 3 and 6 months in the unfavourable group. As shown in Table 4, one of the patient with severe disability GOS at 3 months improves to moderate disability GOS at 6 months. Another patient whom has a GOS of moderate disability at 3 months improves to GOS of good recovery at 6 months. Otherwise there was no significant changes noted upon McNemar test, *P* = 0.368.

Univariate logistic regression analysis was done to predict the outcome of mild head injury patients managed in GSU using the aid of teleneurosurgery, as shown in Table 5. Malay ethnicity (*P* = 0.021) has a 6 times the odds of having favourable outcome compare to other race when other factors were not adjusted. An increase in 1 unit of GCS affects the outcome by the odds of 2.3 times for a favourable outcome (*P* = 0.024) when other factors were not adjusted. No other factors seem to significantly affect the outcome.

## Discussion

In this modern era of medicine, injury ranks among the highest cause of hospital admission. According to Health Facts, Ministry of Health (MOH), Malaysia, injury was documented as the fifth commonest cause of hospitalisation in MOH hospitals in the year 2014 (7.86%) and 2015 (7.64%) (11, 17, 18). One of the major international causes of morbidity, mortality and socioeconomic costs were attributed to head injury patients (19, 20). The vast majority of patients that suffers from head injury are young adults (5, 20). Based on the National Trauma Database 2009, the younger age group ranging between 15–34 years old (56.6%) was at the highest risk of major trauma. There were 92.5% patients with major trauma underwent CT brain imaging and 63.61% of those with major trauma needed to undergo cranial surgical intervention (21).

With the vast increase in traumatic brain injury cases every year, the demand for neurosurgical services have been increased exponentially. Due to the short supply of NSU, which are only provided in certain tertiary centre hospitals, and the great demand for neurosurgical services, there is a necessity for head injury patients to be managed in the peripheral hospitals under the care of GSU (5). With the help of teleneurosurgery, the information exchange between general surgeons and the neurosurgeon in the form of clinical data and scan images were used in order to manage the patients in the peripheral hospital. Studies have shown that teleneurosurgery reduces the unnecessary transfer of patients and medical costs (2).

Study by Fabbri et al. have shown that the practice of managing head injury patients with initial CT scan showing non-surgical lesion in the primary hospital under GSU does not put the patient at higher risk with the aid of telemedicine facilities (22). In this study, we have noted that 2.79% of patients with mild head injury, needing a delay transfer from the primary hospital to NSU HSAJB for further evaluation or neurosurgical intervention. This result is comparable with the study by Klein et al., that shows 3% of their study population needing a delay transfer to a NSU (13). There is 3.07% of the study population falls into the unfavourable group with 10 (89.9%) was contributed by patients needing a delay transfer, with one patient was discharge from the primary hospital with a lower GCS from presentation. The reason to classify those patients needing a delay transfer to NSU as unfavourable outcome is to determine the percentage of failure in using teleneurosurgery as an alternative tool in remotely managing head injury patients.

There are studies showing that mild traumatic brain injury may show lower rate of good GOS recovery when complicated with focal brain lesion and or with depressed skull fracture (10, 23). Patients with GCS of 15, have a risk of poorer outcome if needing surgical intervention comparatively with those treated conservatively. A delay in diagnosis in these patients were suggested to have influenced this outcome noted (23). The difference between the GOS at 3 and 6 months for the unfavourable patients was not statistically significant (*P* = 0.368) in our study. This is most likely due to the limitation of the study, having a small sample size especially those in the unfavourable group. Future study with a bigger sample size could be done in order to address this issue.

Mild head injury patients in this study which were managed in GSU in the primary hospital are considered to have low potential for deterioration. In any instance of deterioration happened, the transfer to NSU in a timely manner was considered safe (13). GCS has been shown to be associated with patients’ outcome and mortality (5, 13). Our study has shown that Malay ethnicity (*P* = 0.021) and GCS at referral (*P* = 0.024), significantly affect the outcome, when other factors were not adjusted. Otherwise, this result was not reproducible when other factors were added into the equation. This is likely to be reflected due to the small sample size in this study.

The ever evolving of data transfer technology, also have a global impact in our medical fraternity. The prospect of managing patients remotely with the help of teleneurosurgery in a non-neurosurgical centre is an appealing prospect. Otherwise the outcome of patients managed using this model need to be further evaluated in order to determine the effectiveness and the safety of the model practiced. In the future, a bigger sample volume involving multiple centres practicing this model of management could be useful in order for us to fully understand the impact of teleneurosurgery for the patients, socioeconomic impact and neurosurgery practice.

## Conclusion

Malay ethnicity and GCS on referral were noted to be significant factors in determining the outcome of mild head injury patients managed in non-neurosurgical centres in Johor state using the help of teleneurosurgery. The percentage of failure in utilising this model of practice is relatively low, 3.06%.

## Limitation and Future Recomendations

Limitation of the study is mainly due to the small sample size in the unfavourable group as compared to the favourable group. A prospective and multicentric model study with a bigger sample size is proposed in order to address the limitation encountered in this study.

## Figures and Tables

**Table 1:** Canadian CT head rule : patients with minor head injury with at least one of the following

A. High risk (for neurological intervention) GCS score < 15 at two hours after injury Suspected open or depressed skull fracture Any sign of basal skull fracture (haemotympanum, ‘racoon’ eyes, cerebrospinal fluid otorrhoea/rhinorrhoea, Battle’s sign) Vomiting > two episodes Age > 65 years
B. Medium risk (for brain injury on CT) Amnesia before impact > 30 min Dangerous mechanism (pedestrian struck by motor vehicle, occupant ejected from motor vehicle, fall from height > three feet or five stairs)

**Table 2:** Characteristics of study population (*n* = 359)

	*n*	(%)
Gender:		
Male	277	77.20
Female	82	22.80
Age, mean (SD)	45.39 (20.23)	
Ethnicity:		
Malay	217	60.45
Chinese	91	25.35
Indian	18	5.01
Foreigner	28	7.80
Others	5	1.40
Mode of referral		
Teleconference	179	49.86
E-mail	180	50.14
Referral Hospital		
Hospital Muar	105	29.25
Hospital Batu Pahat	98	27.30
Hospital Sultan Ismail	83	23.12
Hospital Segamat	38	10.58
Hospital Kluang	35	9.75
Referral GCS, mean (SD)	14.52 (0.72)	
13	49	13.65
14	76	21.17
15	234	65.18
Discharge GCS, mean (SD)	14.89 (0.78)	
3	1	0.28
8	1	0.28
13	2	0.56
14	16	4.46
15	335	93.31
Radiological Dx		
EDH	42	11.7
SDH	115	32.0
CONTUSION	63	17.5
SAH	43	12.0
NO ICB	96	26.7
Transfer		
Manage In GSU	349	97.21
Delay Transfer	10	2.79
GOS at 3 months		
Death	1	0.28
Severe Disability	2	0.56
Moderate Disability	2	0.56
Good Recovery	319	88.86
Missing Data	35	9.75
GOS at 6 months		
Death	1	0.30
Severe Disability	1	0.30
Moderate Disability	2	0.60
Good Recovery	312	86.90
Missing Data	43	12.00
Duration of stay, mean (SD)	2.16 (1.19)	
Outcome		
Favourable	348	96.94
Unfavourable	11	3.06

SD = Standard deviation

**Table 3:** Characteristics of study population by favourable and unfavourable outcome (*n* = 359)

	Favourable		Unfavourable	

	*n*	(%)	*n*	(%)
Gender:				
Male	268	96.75	9	3.25
Female	80	97.56	2	2.44
Etnicity:				
Malay	214	98.62	3	1.38
Chinese	87	95.60	4	4.40
Indian	16	88.89	2	11.11
Foreigner	26	92.86	2	7.14
Others	5	100.00	0	0.00
Referral GCS:				
13	46	93.88	3	6.12
14	71	93.42	5	6.58
15	231	98.72	11	1.28
Radiological Dx				
EDH	40	95.2	2	4.8
SDH	112	97.4	3	2.6
CONTUSION	59	93.7	4	6.3
SAH	42	97.7	1	2.3
NO ICB	95	99.0	1	1.0

**Table 4:** GOS at three and six months for unfavourable group

	GOS at three months	GOS at six months
Good Recovery	6	7
Moderate disability	2	2
Severe disability	2	1
Vegetative state	0	0
Death	1	1
Total	11	11

McNemar test, *P* = 0.368

**Table 5:** Univariate logistic regression analysis

	B(SE)	Crude OR (95% CI)	*P*-value
Gender			
Male	−0.295 (0.792)	0.744 (0.16–3.52)	0.709
Female		1	
Age	0.004 (0.016)	1.004 (0.97–1.03)	0.826
Race			
Malay	1.804 (0.781)	6.071 (1.32–28.03)	0.021
Chinese	0.616 (0.730)	1.851 (0.44–7.74)	0.399
Others		1	
Referral GCS	0.816 (0.361)	2.262 (1.12–4.59)	0.024
Radiological Dx			
EDH	−1.558 (1.239)	0.211 (0.02–2.39)	0.209
SDH	−0.934 (1.163)	0.393 (0.04–3.84)	0.422
CONTUSION	−1.863 (1.130)	0.155 (0.17–1.42)	0.099
SAH	−0.816 (1.426)	0.442 (0.03–7.24)	0.567
No ICB		1	
Duration of stay	−0.063 (0.409)	0.939 (0.42–2.09)	0.878

B = Regression coefficient SE = Standard error OR = Odd ratio
